# Epidemiological Characteristics of Cancer in Elderly Chinese

**DOI:** 10.5402/2012/381849

**Published:** 2012-12-30

**Authors:** Xiao Nong Zou, Xia Wan, Zhen Dai, Gong Huan Yang

**Affiliations:** ^1^Office of Cancer Prevention and Control, National Cancer Center, Cancer Institute & Hospital, Chinese Academy of Medical Sciences, 17 Panjiayuannanli, Chaoyang District, Beijing 100021, China; ^2^Department of Cancer Epidemiology, Peking Union Medical College, Cancer Institute & Hospital, Chinese Academy of Medical Sciences, 17 Panjiayuannanli, Chaoyang District, Beijing 100021, China; ^3^Institute of Basic Medicine, Chinese Academy of Medical Sciences, 5 Dongdansantiao, Dongcheng District, Beijing 100005, China

## Abstract

*Background*. Population of elder Chinese has been increasing, but the pattern and trend of cancer in that population was rarely reported. *Methods*. Mortality rates for cancer of all sites and of the site specific of the overall and elderly Chinese from 2004 to 2005 were estimated. The age structure of world population was used to observe the changes in the age-standardized mortality rates from 1975 to 2005 using the data from the national death surveys, Disease Surveillance Points, and cancer registries in China. *Results*. The mortalities among the elderly Chinese were 782.12 per 100,000, substantially higher than those of the people less than 60 years old. The mortalities for cancers of lung, stomach, liver, and esophagus in elderly population showed great increase compared to younger ages. Stomach cancer ranked as the second most common cancer following lung cancer in the elderly, and those two malignancies had similar mortality rates in male elderly, while in female, it ranked as first, surpassed lung cancer. Consistent decreased trends of M/I ratios of cancer were observed in all age groups. *Conclusion*. Strategies in cancer prevention and cost-effective preventive intervention should be highly considered and strongly implemented among the elderly Chinese.

## 1. Introduction

The recent national population survey showed that the elderly proportion has substantially increased in China in the last decades. In 2011, the number of Chinese in ages of 60 years and older reached at 177 million, up to 13.26% of the residents of mainland China, an increase of 2.93% compared to the same ages in 2000, according to the report of the National Statistics Bureau [1]. The health statistics of the Ministry of Health showed that malignant neoplasm ranked as first cause of deaths among both urban and rural residents of China in that time [2]. Lung cancer was the leading cancer cause of deaths in 2004-2005 resulted from rapid increasing of the mortality in the last thirty years [3–6]. 

However, there were few reports specifically on the patterns and the trends of cancer among the elderly Chinese, and the analyses were mainly limited for a city and county, or the cases in hospitals [7–9]. It is not clear for the characteristics of cancer in the elderly Chinese at national level. In the current paper, we analyzed the data from national death surveys, disease surveillance, and the cancer registry to describe the epidemiological characteristics and estimate the trends of cancer mortality in elderly Chinese.

## 2. Material and Methods

### 2.1. Definitions and Data

Data used for analysis in this study were based on the statistical reports from National Death Surveys conducted in China for the periods of 1973–1975 and 2004-2005 [3–5], the Disease Surveillance Point (DSP) for the years from 1991 to 2000 [10], and the annual reports of national cancer registry [11–13]. The 1973–1975 death survey also called the “national mortality survey” covered 2,392 counties or about 850 million persons and included information from family members and medical records on causes of death by age and sex. Death causes were classified into infectious diseases, pneumonia, tuberculosis, cancer, including lung cancer and stomach cancer, cerebrocardiovascular diseases, chronic respiratory diseases, chronic bronchitis, hepatocirrhosis, and other noncommunicated diseases and injury, which was linked to ICD by setting mapping table of ICD. The Disease Surveillance Points (DSP) system for causes of death 1991–2000 covered 10 million persons in 145 locations in all provinces by multiple-stratified random sampling. This nationally representative sample reflected regional population distributions, urban and rural areas, age and sex, and eastern, middle, and western regions. Following new legislation in 1992, a uniform cause of death certification procedure is now in place for institutional deaths in China, based on the international format of the medical certificate of cause of death. For home-based deaths, causes of deaths are determined based on information provided by family members with or without documentary medical evidence. Several studies have validated the causes of death reported by the DSP system [14–19]. Every 3 years, a survey to estimate underreporting is conducted covering 5 percent of the surveillance population, and mortality estimates are adjusted accordingly. The data bank from DSPs included more than half million deaths with causes classified on ICD-9 each year.

DSP system was expanded since 2001, covered 71.4 million population, each point of which expanded to whole county, or district, covering 300,00–700,000 populations. In order to check the quality of reporting data of the expanding DSP system, National death survey was carried out in 2006, recollected death cases during 2004-2005 in expanded DSP system. The total 868,484 death cases were collected with household inquiry. The verbal autopsy instrument was used for death cases without hospital diagnosis [17–19]. About 6.44 percent death cases were diagnoses with VA instruments. The classification of death cause was according to the ICD-10. The DSP data during 2004-2005 were the same with national survey [3].

The number of cancer registries in China was increasing, and the quality of data was improving, since the National Central Cancer Registry was established in 2002. There were only 12 cities/counties that were included in the data analysis for the period of 1993–1997 in 2002, while in 2008, the data from 41 cities/counties were analyzed to generalize the incidence and mortality of cancer among a total of 66,138,784 people in China [11, 12]. We used the rates of incidence and mortality of cancer reported for two periods of time [11, 12] to calculate these M/I ratios by age.

We defined that the cancer in the elder population is the occurrence of cancer or death in people aged 60 and over. To verify the increasing and decreasing trend of cancer, age-standard death rates from 1973 to 2005 were computed based on WHO standardized population structure [20].

## 3. Result

### 3.1. The Mortality Pattern of Cancer in Elder Chinese

**Figure 1:**
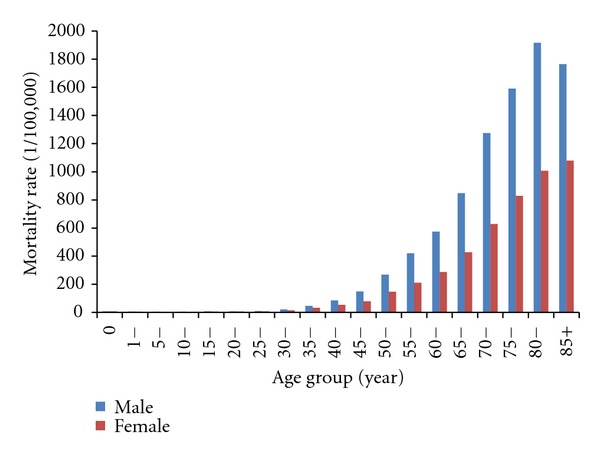
The age-specific death rate of cancer in China, 2004-2005.

The age-specific death rates of the cancer for all sites in 2004-2005 are shown in Figure 1. The mortalities among the elderly Chinese were 782.12 per 100,000 (1021.21 per 100,000 in men and 555.11 per 100,000 in women), substantially higher than those of the people less than 60 years old. There were 65.49% (65.01% in men and 66.34% in women) of the cases dead from cancer in ages of 60 and above, and the rates are higher for men than women. It was also found higher mortalities in the urban people aged 60 and over (1,086.95/100,000 in men and 593.67/100,000 in women) than those of rural (983.89/100,000 in men and 532.70/100,000 in women).

**Figure 2:**
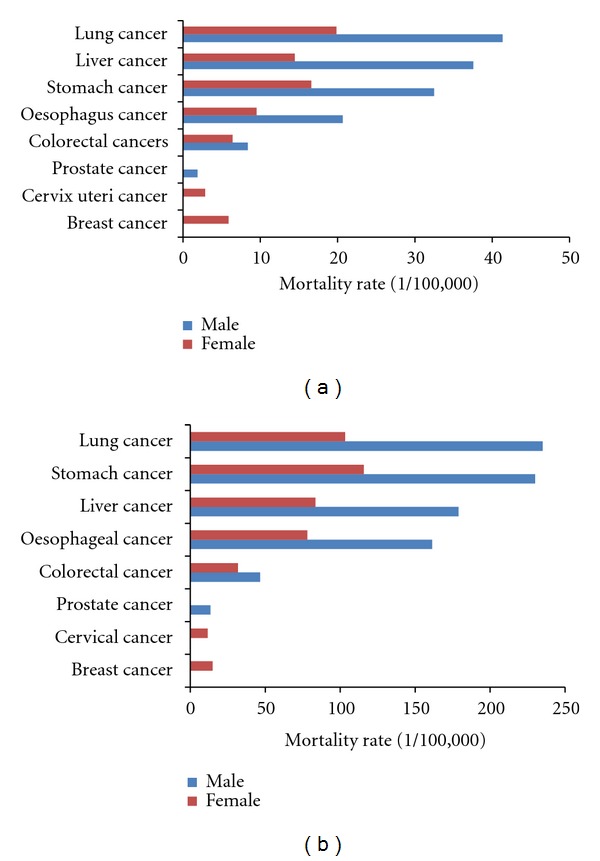
The site-specific mortality rate of cancer for whole population (a) and for people aged 60 and above (b) in China, 2004-2005.

The mortalities for common cancers among the whole population and the elderly population in 2004-2005 were shown in Figures 2(a) and 2(b). Though four leading malignant diseases included cancers of lung, stomach, liver, and esophagus, the age-standardized mortality rates in elderly population were found as 5 to 8 times of the rates in the whole population. In the elderly, stomach cancer ranked as the second most common cancer following lung cancer in male, and the mortalities of those two cancers had the similar level. While in female, stomach cancer ranked as the first with a high age-standardized mortality of 12% surpassed the cancer of lung.

### 3.2. The Changes of the Mortality of Major Cancers in Elder Chinese

**Figure 3:**
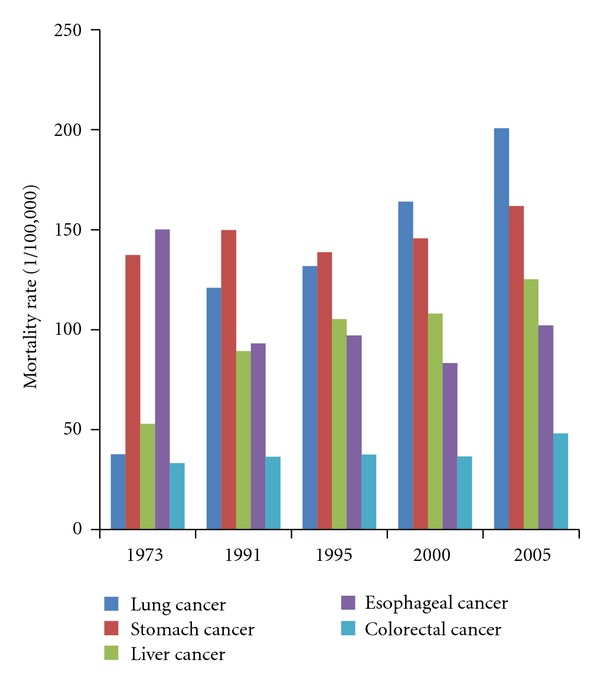
The site-specific standardized mortality rates of cancer for people aged 60 years and older in China from 1973 to 2005.

In 1970s, the cancers of stomach and esophagus were the two most common cancers in elderly population of China, as shown in Figure 3. While in 1991, the most common cancer was stomach cancer, and the age-standardized mortality of esophageal cancer showed a markedly decrease. Lung cancer became the leading cancer after the year of 2000. The mortality rates for the cancers of liver and colorectal also showed increasing trends.

### 3.3. Ratio of the Mortality to the Incidence and the Estimated Case of Cancer in Elderly People

The ratios of the mortality to the incidence (M/I ratio) of cancer for all sites combined in two time periods were shown in Table 1. In 1993–1997, the M/I ratio for all ages was 0.70, and those were 0.49, 0.57 and 0.79 for the ages in less than 40 years, 40–59 years, and 60 years and older, respectively. These ratios were found consistent higher in men than in women of the age specific groups. While in 2008, consistent relatively low ratios were found for the overall population (0.62) as well as for each age groups, that is, 0.31, 0.46, and 0.74 for ages of less than 40 years, 40–59 years, and 60 years and older.

## 4. Discussion

In the current study, we reported that more than 60% of cases of cancer were from Chinese elderly population, and lung cancer leaded the causes of cancer, which was similar to observed in the general population of China [21–24]. Recent studies showed that the major health problems among Chinese population were more likely to be the chronic diseases, including cancers of lung, liver, female breast, and the coronary heart disease, stroke, diabetes, and traffic accidents in the general population of China in 1991–2000 based on our early reports [6]. These emerging burdens of disease were mainly owing to the contribution of nondemographic factors including economic development, popularization of education, and health services, and particularly to the long time exposure to tobacco use [21–25]. The increased mortality rates of lung cancer among elderly Chinese in the last decades were consistent with the observation on the general population and were the most important contributor of the increase of the cancer mortality, and even markedly decreased rates were observed in cancer of esophagus (Figure 3). Consistent increase of lung cancer in the general and elder population could be also considered the delayed effect of active and passive smoking of the Chinese population [25–27]. Lung cancer mortalities in developed countries showed markedly reduction after the prevalence of smoking having been rapidly decreased [28]. These best-buy preventive strategies to prevent cancer by keeping people from smoking should be implemented in all ages of the residents in China, particularly for the health of the elderly. 

Consistent trends of the mortalities were found in both the population of all ages and the elderly population in the last 30 years in this study. Different spectra could be found when analysis conducted for specific site, while lung cancer was the leading cancer in both the all ages and the population age 60 years and older. Unlike the decreased trends observed in the general population [22–25], the mortality of stomach cancer, rather than liver cancer, following cancer of lung, ranked as the second of cancer deaths in the elderly population. Moreover, higher M/I ratios could be found in the elderly than those in the population younger than age of 60, which indicated a shorter survival living time in those cases after diagnosed as cancer. These may be due to the more cases in the elderly been diagnosed at late stages of the diseases and had poor progress than the persons at younger ages.

Even though the survival of the cancer patients had increased in the past 10 years, the survival in the elderly was still low, as shown from the high M/I ratios in Table 1 of this paper. Those mean shorter time of survival, later diagnosis, and poorer response to the treatment in the elderly than the young population in China. Different cancer spectra could be one of the reasons for the consistent high M/I ratios in male than female. Female breast cancer had better survival, while the cancers of the lung, liver, and stomach had poor survival, and the rates were much higher in male than in female [22–24, 26, 27].

China is experiencing a rapid transition movement from low- to middle-income levels in the social and economic development. The number of elderly people will be continuously increasing to reach 182 million in 2020 from 177 million in 2011 if the 10-year aging rate of 2.93% keeps the same. Then, the numbers of cancer deaths and new cases of cancer in elderly Chinese will be estimated as 1.44 million and 1.82 million annually, based on our statistics. Strategies in cancer prevention and control should be comprehensively investigated, and specific health promoting programs and cost-effective preventive intervention should be highly considered and strongly implemented among the elderly population. In addition to the progress of the aging population and incoming of the tobacco hazards, encouragement to quit the bad lifestyle and prevention from infection with hepatitis virus, *helical pylori,* and other carcinogenic agents would also make contribution to the epidemic cancer in China. The social and economical determinants should also be considered in developing programs to assure continuing development of this developing country to follow the achievement gained in the USA, UK, and Finland, where cancer mortality had been greatly reduced accompanying the great investment in primary prevention of cancer.

## Figures and Tables

**Table 1:** Ratios of mortality to incidence of cancer in 1993–1997 and 2008.

	Male and female			Male			Female		
	Cases	Deaths	M/I	Cases	Deaths	M/I	Cases	Deaths	M/I
1993–1997									
All ages	230023	161607	0.70	132472	99385	0.75	97552	62222	0.64
<40 yr	18694	9083	0.49	9316	5579	0.60	9378	3504	0.37
40–59 yr	62062	35248	0.57	33171	21841	0.66	28891	13407	0.46
≥60 yr	149267	117276	0.79	89984	71966	0.80	59282	45311	0.76

2008									
All ages	197833	122136	0.62	110077	76062	0.69	87756	46074	0.53
<40 yr	11667	3626	0.31	4814	2071	0.43	6853	1555	0.23
40–59 yr	67030	30598	0.46	33893	19757	0.58	33137	10841	0.33
≥60 yr	119136	87912	0.74	71370	54234	0.76	47766	33678	0.71
